# Telomere length declines with age, but relates to immune function independent of age in a wild passerine

**DOI:** 10.1098/rsos.212012

**Published:** 2022-04-27

**Authors:** Michael J. Roast, Justin R. Eastwood, Nataly Hidalgo Aranzamendi, Marie Fan, Niki Teunissen, Simon Verhulst, Anne Peters

**Affiliations:** ^1^ School of Biological Sciences, Monash University, Victoria 3800, Australia; ^2^ Groningen Institute for Evolutionary Life Sciences, University of Groningen, Nijenborgh 7, 9747 AG Groningen, The Netherlands

**Keywords:** senescence, quality, ageing, constitutive, immune, avian

## Abstract

Telomere length (TL) shortens with age but telomere dynamics can relate to fitness components independent of age. Immune function often relates to such fitness components and can also interact with telomeres. Studying the link between TL and immune function may therefore help us understand telomere–fitness associations. We assessed the relationships between erythrocyte TL and four immune indices (haptoglobin, natural antibodies (NAbs), complement activity (CA) and heterophil-lymphocyte (HL) ratio; *n* = 477–589), from known-aged individuals of a wild passerine (*Malurus coronatus*). As expected, we find that TL significantly declined with age. To verify whether associations between TL and immune function were independent of parallel age-related changes (e.g. immunosenescence), we statistically controlled for sampling age and used within-subject centring of TL to separate relationships within or between individuals. We found that TL positively predicted CA at the between-individual level (individuals with longer average TL had higher CA), but no other immune indices. By contrast, age predicted the levels of NAbs and HL ratio, allowing inference that respective associations between TL and age with immune indices are independent. Any links existing between TL and fitness are therefore unlikely to be strongly mediated by innate immune function, while TL and immune indices appear independent expressions of individual heterogeneity.

## 1. Introduction

Telomeres are structurally protective ends to linear chromosomal DNA strands [1] which shorten during DNA replication and repeated cell divisions [2]. As such, telomere length (TL) tends to decline with age and more rapidly during growth phases [3]. Telomeres are also susceptible to oxidative damage [4], and exposure to oxidative stress can accelerate telomere attrition (review: [5]; but see [6]). Both intrinsic and extrinsic stressors (i.e. perturbations to homeostasis such as infection, reproductive effort and adverse environments) have been linked to telomere attrition [7], with metabolic, hormonal and/or immune mechanisms hypothesized to be involved [8–10]. Given that longer telomeres are often related to longer lifespans or greater lifetime reproduction in wild organisms independently of age [11–13], individual TL could be a useful biomarker of individual quality (fitness potential) reflecting resilience to cellular ageing and oxidative processes. On the other hand, the rate or extent of telomere loss over time might better predict future fitness [14–16], possibly by reflecting the severity or cumulative effects of stressors [17,18]. Moreover, the adaptive significance of changes in TL might depend on the trade-off between different components of fitness (e.g. reproduction versus longevity [15,19]), or even hormetic signalling [20]. A complete understanding of telomere dynamics and potential implications for fitness remains unclear.

Immune function is crucial for fitness in the face of ubiquitous parasite pressure, directly via morbidity or mortality, or indirectly via fitness-related traits (e.g. sexual ornaments, social dominance; [21] and references therein). Because telomere attrition can induce cellular senescence and generate dysfunctional secretory profiles [22], TL variation in immune cells, or cells producing humoral immune components, could have significant fitness implications. In humans for example, shorter leucocyte TL is related to higher susceptibility to experimental infections [23], higher mortality rates [24] and most recently with adverse COVID-19 patient outcomes [25,26]. Across a range of vertebrate taxa, overall relationships between TL and mortality risk are evident ([13] though mostly arising from cross-sectional studies, [2]); whether immune function facilitates such telomere–fitness links is less evident. Interactions between telomere attrition and immune function are potentially complex, as immune activation might influence telomere attrition as a long-term cost of infection [27]. Interestingly, repeated infections that induce telomere attrition [28–30] could possibly create a feedback loop that accelerates immunosenescence and mortality risk, where the remaining TL represents some individual quality and resilience to future infection. Investigating relationships between TL and immune function is therefore necessary to better understand plausible mechanistic links between TL and fitness, and where possible, through experimental designs manipulating immune function and telomere dynamics in wild organisms, while monitoring fitness outcomes. Furthermore, a longitudinal framework is necessary to account for relationships that may exist at the individual and population levels.

To assess the relationships between TL and immune function, we combined longitudinal telomere and immune datasets collected during long-term individual-based studies of wild purple-crowned fairy-wrens (*Malurus coronatus*). Using a large sample, we assessed at both the individual and population levels how TL of red blood cells (RBCs) relates to indices of innate immune function: haptoglobin-like protein (Hp; or the analogous PIT54 in some avian species), natural antibodies (NAbs), lytic complement activity (CA) and heterophil-lymphocyte (HL) ratio. While functionally diverse, these indices comprise important aspects of primary immune defences involved in pathogen recognition and elimination. Because NAbs, CA and Hp are all humoral immune components secreted from immune and other somatic cells [31,32], we hypothesized that TL-associated dysregulation of secretory profiles will impact the production of these components. These three immune indices are constitutively maintained regardless of infection history ([21]; cf*.* Hp during acute phase response), thus we did not expect the direction of associations with TL to represent an individual-level cost of infection. Rather, we predicted that with decreasing TL within individuals, the production of these immune components would decline. HL ratio is a composite metric of cells involved in innate and adaptive immune functions, which can also function as an indicator of chronic stress [33]. Because stressor exposure has been consistently linked to declines in TL in humans and animals (though mostly cross-sectional studies—see meta-analyses [7,34]), we predicted HL ratio to correlate negatively with TL, due to both parameters responding to stressor exposure. An alternative hypothesis predicting a (causal) negative relationship between TL and HL ratio is that the leucocyte-producing thymus, which involutes showing signs of cellular senescence in almost all vertebrates [35], stops producing circulating leucocytes (naive T-cells) and increases the HL ratio.

**Figure 1.**
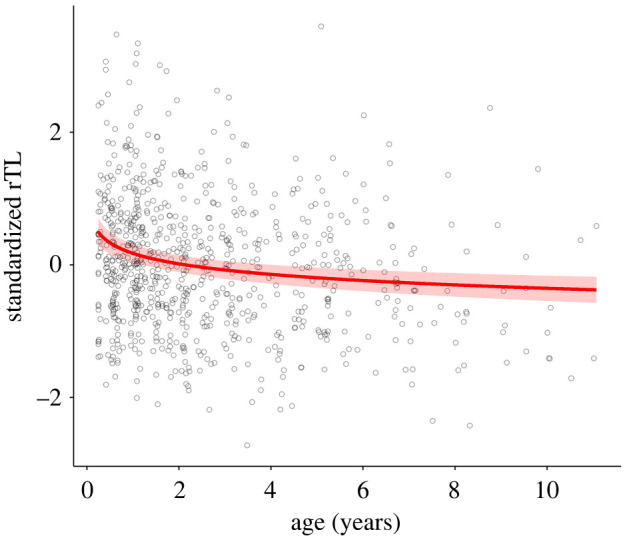
Declining TL with increasing age in purple-crowned fairy-wrens. A significant nonlinear relationship between relative telomere length (rTL) and age as shown by generalized additive mixed models (GAMMs) can be well approximated by log age (electronic supplementary material, table S2). Fitted values show standardized rTL predicted by log age in a linear mixed model (LMM), and back-transformed to the original age scale, with the ribbon showing 95% confidence intervals, incorporating uncertainty from all fixed effects in the LMM.

Individuals in this population are of known age, and in previous work examining these four immune indices, we found age-related changes in NAbs and HL ratio at the population level, but without clear within-individual immunosenescence [36]. We also have an expectation that telomeres will show age-related shortening, most rapidly in early life [12,14]. If strong relationships between age and TL are present at either the individual or population levels, we might expect similar telomere-related changes in immune function to those we observed previously for age. Nevertheless, TL integrates information not only about age, but also individual quality and/or stressor exposure. Consequently, we employ here a similar statistical framework to Roast *et al*. [36] to longitudinally assess how TL relates to these indices, while controlling for independent age-related changes in immune function. In this framework, effects of TL on immune indices should reflect integrated information like individual quality beyond age-related telomere attrition. With this longitudinal study, we expect to contribute to an understanding of telomere-immune relationships across the lifespan, which is currently minimal in wild populations (figure 1, [27]).

## 2. Methods

### 2.1. Study population, capture and sampling

Purple-crowned fairy-wrens are resident birds inhabiting riparian vegetation in tropical savannahs of northern Australia. The species forms cooperatively breeding social groups that occupy distinct territories in suitable habitat [21,37]. Our study population is located at the Australian Wildlife Conservancy's Mornington Wildlife Sanctuary (126.1° E, −17.5° N), along 15 km of Annie Creek and Adcock River. All individuals have been uniquely colour-banded and routinely monitored since 2005 as part of a long-term research project, providing detailed life-history profiles of each individual from hatching or immigration into the population, until dispersal or mortality [21]. A tropical, riparian and social environment exposes purple-crowned fairy-wrens to numerous pathogens, including confirmed endoparasitic infections of *Haemoproteus* sp., *Plasmodium* sp., *Trypanosoma* sp., *Coccidia* sp., and microfilarial nematodes [38].

Blood sampling was undertaken during two separate field sampling periods each year (mid-April to mid-June; mid-October to late November) from April 2012 to June 2017. During these 11 field sampling periods, a total of 796 blood samples were collected from 331 individuals from which both TL and at least one immune index have been quantified for this study (relative telomere length (rTL) and Hp, *n* = 11 sampling periods, 2012–2017; NAbs and CA, *n* = 7, 2012–2013 and 2016–2017; HL ratio, *n* = 9, 2012–2014 and 2016–2017). From each blood sample, the complete suite of immune index measurements could not always be made in addition to TL, thus final sample sizes for each immune index differ, but are subsets from within this total (specific details of final sample sizes are reported below; electronic supplementary material, figures S1 and S2). Upon capture in mist-nets, birds were transferred to holding bags until blood was sampled (median holding time = 23 min, s.d. = 19.6 min) to mitigate handling stress that can influence immunological indices [39,40]. Up to 100 µl blood was collected by brachial venipuncture into heparinized capillary tubes that were sealed and stored on ice. The same day, samples were centrifuged at 16 060*g* for 5 min to obtain RBC and plasma fractions. RBCs were separated and placed in excess absolute (AR grade) ethanol, before continued storage at 4°C ready for DNA extraction. Plasma fractions were frozen at −20°C before transfer within eight weeks to continuous storage at −80°C until use in immune assays; the immune indices we analysed are resilient to multiple freeze–thaw cycles and longer-term storage [41]. Blood smears were also created at capture using the wedge-pull method with whole blood [42], before air-drying and fixation in absolute (AR grade) methanol for at least 15 min.

### 2.2. Telomere analyses

DNA was extracted from erythrocytes using a QIAamp DNA kit and QIAcube HT instrument (Qiagen) with protocol modifications as per Eastwood *et al*. [43]. DNA extracts from all samples were assessed using a NanoDrop (ND-1000) for purity and concentration, and run on a 1% agarose gel (100 V, 30 min) to check DNA integrity. rTL was quantified using a qPCR method [44] that has been modified and validated for use in *M. coronatus* [12,43]. The qPCR reaction mixes were automatically prepared using an EpMotion 5075 on 96-well plates. In each well, the reaction mix totalled 25 µl, including: 12.5 µl of SYBR Green I (Roche); 300 nM of both normalizing control gene (glyceraldehyde-3-phoshate dehydrogenase, GAPDH; [45]) primers or 400 nM of both telomere primers (Integrated DNA Technologies; [12]); 10 ng of sample DNA or 10 ng of an inter-plate control sample made up of DNA from multiple *M. coronatus* individuals. All samples and controls were run in duplicate on each plate, for both telomere and GAPDH amplification. Reactions were conducted in a LightCycler 480 (Roche) with the following protocols: telomere (95°C for 15 min, followed by 35 cycles of 15 s at 95°C, 30 s at 56°C, 30 s at 72°C) and GAPDH (95°C for 15 min, followed by 40 cycles of 15 s at 95°C, 30 s at 60°C, 30 s at 72°C). Correct product amplification and qPCR quality were assessed for both reactions by visualizing melt-curves and the no-template control (nuclease free water). qPCR efficiencies were within 100 ± 15% and were calculated from a 10-fold serial dilution starting at 40 ng of DNA from the control sample. Intra-assay Cq repeatabilities were 0.91 (telomere) and 0.97 (GAPDH). The rTL was calculated using equation 1, in [43]. The repeatability of rTL was high at both inter-assay (0.85) and inter-extraction (0.88) levels.

### 2.3. Immune indices

Four commonly assessed innate immune indices that are important for primary immune defences and infection detection were quantified: (i) haptoglobin, (ii) NAbs, (iii) CA and (iv) HL ratio. Detailed methodological descriptions are found in Roast *et al*. [46], and here reported only in brief. First, haptoglobin (Hp; or PIT54 functional analogue in some avian species) scavenges and binds to the toxic and oxidative haem groups produced during infection or inflammation by damaged cells [47,48]. Baseline Hp—predictive of acute phase levels during an immune response [49]—was analysed in a colorimetric assay following [46]. Corrections were applied for plasma turbidity (using pre-scans at 630 nm) and redness (using 450 nm absorbance values in statistical models; this was necessary as redness affected Hp values at 630 nm; electronic supplementary material, tables S4–S7a). Inter-plate variation was assessed using a rabbit plasma standard in triplicate on each plate (coefficient of variation, CV = 0.24, *n* = 25 plates); samples were plated in duplicate. Below the optical saturation threshold for the assay (1.25 mg ml^−1^), samples formed a normal distribution (mean = 0.62 mg ml^−1^, s.d. = 0.27). Second, NAbs are non-specific antibodies that are produced prior to any antigenic exposure and are crucial for binding to novel antigenic components on pathogens [50]. Third, CA of the lysis complement system consists of sequentially activated lytic proteins, which proceed to lyse and eliminate pathogens and foreign cell components [31]. Both NAbs and CA were quantified using the same haemolysis-haemagglutination assay [51], following modifications in [46], with inter-plate CV = 0.13 and CV = 0.11 respectively. Fourth, the HL ratio consists of heterophils that perform phagocytosis and bactericidal activity integral to cellular innate immunity [52], and lymphocytes that secrete antibodies crucial to the adaptive immune response [53]. Additionally, the HL ratio is an indicator of chronic stress [33], but can also be predictive of immune responsiveness [54]. The HL ratio was counted per 100 leucocytes on May-Grünwald-Giemsa-stained thin blood films [42].

### 2.4. Statistical methods

All statistical analyses were performed using R software v. 3.6.3 [55]. We first assessed the relationship between rTL and age in our dataset. All rTL values (*n* = 796 from 331 individuals) were Z-transformed by subtracting the mean and dividing by the standard deviation. We fitted standardized (normally distributed) rTL values as the response variable in a generalized additive mixed model (GAMM) containing age fitted as a smooth term, in addition to a fixed effect ‘Storage time’—the time in days between initial capture and DNA extraction for telomere analyses [56], and random effects ‘qPCR run’—qPCR processing batch, and ‘individual ID’ to control for repeated measures among individuals. ‘Sex’ was unimportant for TL [12,14], while a ‘Field season’ random effect seemed to explain the same variance as ‘Storage time’ (given that DNA was batch extracted after all field seasons) and therefore was not included in the model. This model showed a significant nonlinear relationship between rTL and age, with a decelerating effect of age on rTL (electronic supplementary material, table S1). We later used rTL as an explanatory term in models, with age as a controlling covariate, and each immune index as a response. To adequately capture the nonlinear covarying relationship between age and rTL in the structure of later models, we assessed whether natural-log-transformed (log) age was a good approximation of the nonlinear relationship between rTL and age. Similar to the GAMM, we fitted a linear mixed model (LMM; using lmerTest package; [57]), but with a log age fixed effect rather than an age smooth function. Using R^2^, AIC, age effect sizes and graphical representations from these two models, we judged that log age approximates well the nonlinear relationship between rTL and age (electronic supplementary material, table S2); we therefore used log age as a covariate in further modelling.

For each immune index, the final number of samples that had an associated rTL measurement was for Hp, *n* = 589 from 297 individuals; NAbs and CA, *n* = 477 from 267 individuals; HL ratio, *n* = 485 from 257 individuals. These samples are subsets of the data used in [36] for which TL measurements were also available. Samples include both single and multiple repeated measures of individuals with up to seven measures per individual for Hp, and up to five repeated measures for all other immune indices (electronic supplementary material, figure S2). CA titre scores were natural-log-transformed, HL ratios were square-root-transformed (all observed raw values were greater than 0 and less than 1) to normalize distributions (electronic supplementary material, figure S3). To assess the relationship between individual TL and immune function, we fitted LMMs containing each immune index as a response variable and rTL as an explanatory term. To use rTL as an explanatory term, we first corrected raw rTL measurements for telomere assay-specific methodological error in a separate linear model, fitting ‘Storage time’ and ‘qPCR run’ as fixed effects, and using standardized residuals from this model as corrected rTL values. In LMMs for each immune index response, we fitted as fixed effects corrected rTL values, with log age as a covariate, in addition to sex, ‘time wait’—the holding time between initial capture and blood sampling, ‘time bled’—blood sampling time as minutes past sunrise (to correct for circadian variation in immune indices; [40]), ‘450_dev_’ to correct for within-subject plasma redness (Hp; [49]), and ‘Plate standard’—to control for inter-plate variation (NAbs and CA). As random effects, we included ‘Plate ID’—to control for plate effects (Hp), ‘Scorer ID’—to control for scorer variation (HL ratio), ‘Field season’—to control for variation or batch effects between field sampling periods and ‘Individual ID’—to control for non-independence of repeated measures. Each model was fitted both with and without log age as a covariate which did not affect model outcomes (see electronic supplementary material, table S4 for LMMs without log age). Additionally, to test for nonlinear relationships between rTL and immune indices, a GAMM equivalent to each LMM was created with rTL as a smooth term; however, in each case, no significant nonlinear effects were supported (see electronic supplementary material, table S5 for GAMMs).

Finally, to assess whether relationships between TL and immune indices differed within and between individuals, we applied a within-subject-centring approach [58]. Corrected rTL values were within-subject centred by subtracting the individual's mean from the observation of that individual to give ‘ΔrTL’, representing the within-individual effect (*β*_W_), specifically addressing whether an individual's immune function changes according to its TL. The individual mean corrected rTL value, ‘*μ*rTL’, was assigned to each observation to represent the between-individual effect (*β*_B_), specifically addressing whether the variation in TL among individuals explains variation in immune function. The two variables, ΔrTL and *μ*rTL, were then applied to the previous LMM in place of the single-corrected rTL variable. For individuals with only a single measurement, ΔrTL = 0, and *μ*rTL = the value of that observation.

## 3. Results

### 3.1. Telomere length declines with age

rTL showed a clear nonlinear decline with age (electronic supplementary material, table S1) in addition to large individual variation across ages. This nonlinear decline shows that TL decreases most rapidly in the first year of life and declines more gradually thereafter (table 1 and figure 1).

**Table 1.** LMM shows the effect of age on rTL. Age effect estimates are on a natural-log-transformed scale. The β-estimates (*β*), standard errors (s.e.), degrees of freedom (d.f.), *t*-statistics (*t*) and associated *p*-values (*p*) for fixed effects and variances (*σ*^2^) attributable to random effects, are shown for each model parameter. Italicized shows parameters for which *p* < 0.05. When this log age effect is decomposed into within- and between-individual age effects on rTL, effect slopes are statistically similar for *β*_W_ and *β*_B_ of log age (electronic supplementary material, table S3).

effect	*β*	s.e.	d.f.	*t*	*p*	*σ*^2^
intercept	3.50 × 10^−1^	9.60 × 10^−2^	194.2	3.644	<0.001	—
log age	*−2.30 × 10^−1^*	*4.22 × 10^−2^*	*622.7*	*−5.460*	*<0.001*	—
storage time	*−1.90 × 10^−4^*	*5.71 × 10^−5^*	*706.7*	−*3.322*	*<0.001*	—
qPCR run	—	—	—	—	—	0.082
individual ID	—	—	—	—	—	0.290
(residual)	—	—	—	—	—	0.575

### 3.2. Telomere length and immune indices

**Figure 2.**
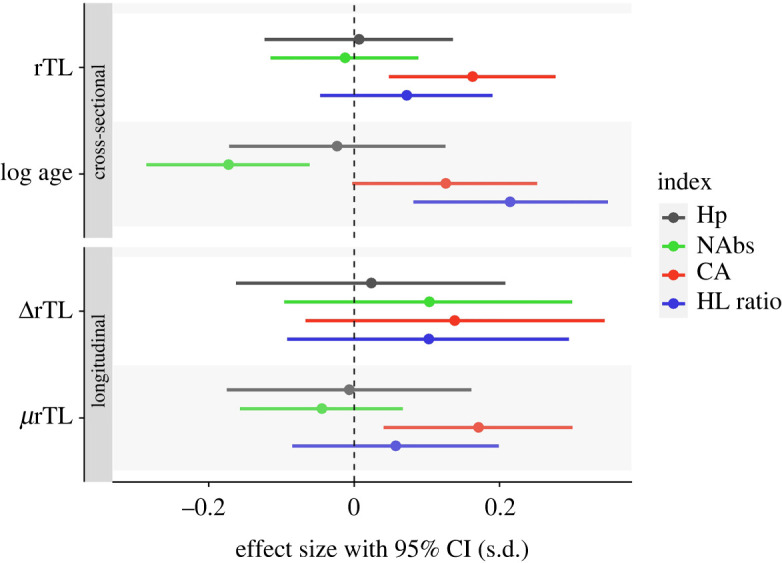
The effect of rTL, natural-log-transformed age, ΔrTL (within-individual) and *μ*rTL (between-individual) on indices of immune function. Each β-estimate is derived from values that were first corrected (as per statistical methods for rTL variables) and standardized prior to inclusion in models. Each β-estimate is then standardized and made comparable among immune index response variables by taking the β-estimate from each model and dividing by the standard deviation of the residuals of the same model minus the respective explanatory term, i.e. the effect is standardized by the deviation in the immune response attributable only to the explanatory term of interest in each model (and some small residual error). The rTL and log age effects presented derive from the cross-sectional LMM, while ΔrTL and *μ*rTL derive from the within-subject-centred (longitudinal) LMM. CA and HL ratio were not back-transformed to their original distributions (from log and square-root, respectively). Bars show 95% confidence intervals calculated using the *confint()* function of the *lmerTest* package, standardized using the same methods as the β-estimates.

rTL did not show any clear relationships either with haptoglobin, NAbs or HL ratio. However, a clear positive linear association between rTL and CA was present, showing that longer telomeres were associated with increased CA (table 2 and figure 2). Controlling for log age did not alter these results (electronic supplementary material, table S4). Significant age effects on immune indices were found only in NAbs (negative) and HL ratio (positive), consistent with previous results in this population using a similar dataset [36].

**Table 2.** LMM β-estimates and 95% confidence intervals of telomere effects on immune function. rTL, natural-log-transformed age, ΔrTL (within-individual) and *μ*rTL (between-individual) β-estimates and 95% confidence intervals are unstandardized and presented in the units of each immune index (not back-transformed for CA and HL ratio). Estimates for the log age covariate are derived from models including rTL, shown fully in the electronic supplementary material, table S6 (as opposed to within-subject-centred models with ΔrTL and *μ*rTL, shown fully in the electronic supplementary material, table S7). Italicized shows 95% confidence intervals that do not contain zero, as calculated using the *confint()* function of the *lmerTest* package.

effect	immune index response variable							
	haptoglobin (*n* = 589)		NAbs (*n* = 477)		CA (*n* = 477)		HL ratio (*n* = 485)	
	*β*	95% CI	*β*	95% CI	*β*	95% CI	*β*	95% CI
rTL	0.001	(−0.016, 0.018)	−0.018	(−0.166, 0.128)	*0.086*	(*0.025, 0.147*)	0.010	(−0.007, 0.027)
log age	−0.003	(−0.023, 0.017)	*−0.250*	(*−0.414, −0.089*)	0.068	(−0.001, 0.135)	*0.030*	(*0.012, 0.050*)
ΔrTL	0.003	(−0.022, 0.028)	0.150	(−0.138, 0.434)	0.074	(−0.036, 0.184)	0.015	(−0.013, 0.042)
*μ*rTL	−0.001	(−0.023, 0.021)	−0.064	(−0.227, 0.097)	*0.091*	(*0.022, 0.159*)	0.008	(−0.012, 0.028)

By within-subject-centring rTL estimates, we examined if and how rTL was related to immune indices within and between individuals, i.e. at the individual and population levels. We found that within individuals, rTL is unrelated to any immune index (table 2 and figure 2). Between individuals, *μ*rTL is significantly related to CA only and unrelated to all other immune indices. The positive between-individual effect of *μ*rTL found only in CA is consistent with the rTL effect found only in CA and suggests the overall relationship between rTL and CA to be mainly driven by between-individual differences. For CA, we further tested the difference between the within- and between-individual rTL slopes, finding that they did not differ significantly from one another (electronic supplementary material, table S8). Therefore, a degree of caution should be applied to interpreting a lack of within-individual effect where a between-individual effect exists. These limitations were explicitly addressed and modelled in [36], with specific consideration to how the structure of this (similar) repeated measures dataset might affect power to detect within-individual effects; this analysis concluded that detection of within- and between-individual effects should be possible except for the smallest effect sizes.

## 4. Discussion

### 4.1. Age-related declines in telomere length

Consistent with findings in most other species [16], we found that TL in purple-crowned fairy-wrens declined with age. This decline decelerated with age, which is consistent with more rapid age-related telomere shortening typically occurring during earlier life stages and/or during organismal growth [3,8]. Calculated from fitted values across the age range we sampled, the instantaneous rate of change in rTL in purple-crowned fairy-wrens was at 6 months, 1, 2 and 4 years = −0.4605, −0.2303, −0.1151 and −0.0576 s.d. year^−1^, respectively. The mean rate of change was −0.0805 s.d. year^−1^, and while not directly comparable, appears a similar magnitude of decline to other small passerines with similar maximum lifespans (akin to TROCrel as *per* table 1b in [59]).

### 4.2. Absent immune- and stress-telomere relationships within individuals

Combining longitudinal data on TL and immune indices, we assessed the relationships between TL and immune function and found that rTL was not significantly related to any immune index within individuals. In other words, individuals that lost more telomeres than average did not also display immune index changes that were larger than average.

We predicted that because humoral immune components are cellular secretions, TL-dependent dysregulation of secretory profiles would negatively impact the production of our humoral immune indices. TL is crucial to maintaining structurally protective telomeric loops which delay cellular senescence and dysregulation. As such, it is possible that the TLs we measured were insufficiently short to breach a threshold that unravels telomeric loops and results in senescent-associated secretory phenotypes (SASPs; [22]). Threshold dynamics alone may be overly simplistic to explain telomeric loop unravelling; however, other impactful factors like DNA breakages and protein levels should work only to distort further the relationship between TL and SASPs [60]. The indirect nature of plausible effects of TL on humoral immune components therefore offers some explanation for the lack of within-individual effects we observed. Additionally for haptoglobin, which functions primarily as a scavenger of reactive oxygen species (ROS) [47], it could be hypothesized that higher baseline concentrations mitigate oxidative damage to telomeres and relate to longer TLs. The relationship between haptoglobin and TL could then operate causally in both directions and possibly exhibit correlations in both directions that result in no net change relationship.

Intra-individual TL variation among tissue types might also explain our results. Both NAbs and (partly) lysis complement proteins are produced by various classes of circulating leucocytes. Immune cells experience higher telomerase activity than other somatic cells as they undergo multiple divisions and renewal [18]. Possibly, high telomerase activity delaying specifically immune cell senescence maintains the production of these particular immune components [61], consistent with our lack of within-individual effects of TL. Furthermore, in mammals, leucocyte TL is obtained from whole blood samples (e.g. [62,63]), where leucocytes are the primary source of DNA in the presence of anucleate erythrocytes. TL measured from blood in birds and other taxa with nucleate erythrocytes derive from a negligible proportion of leucocyte DNA (e.g. in reptiles [64]) and thus does not represent leucocytes, and less so the hepatocytes that produce haptoglobin and (partly) lysis complement proteins. Although we do not know precisely how well TL quantified from avian blood correlates with TL in leucocytes and hepatocytes of interest here, telomere measurements derived from blood correlate well with several other somatic body tissues in zebra finches [65] and adult humans [66], with further evidence from humans suggesting TL correlates among different haematopoietic cell types [67].

Throughout life, individuals are exposed to stressors over various timescales that are thought to manifest at the molecular level as oxidative stress, possibly contributing to telomere attrition (e.g. fig. 3 in [8]; fig. 4 in [7]). As a metric of chronic stress, the HL ratio did not relate to TL within individuals, contrary to expectation and existing literature (e.g. [68]). One reason for this lack of relationship could be a mismatch among timescales, given that TL changes over longer periods (months–lifespans) than HL ratio (hours–months), which in turn is stimulated to change by glucocorticoid stress hormones that change with acute stress (seconds–hours; [33]). Nevertheless, glucocorticoids themselves have been implicated in changes in TL and acute stressors may play a more direct role in inflicting telomere losses [8,69].

### 4.3. Individual quality mediated by complement activity?

By within-subject centring, we demonstrate that between-individual-level differences in mean TL (*β*_B_) were not associated with indices of immune function (NAbs, Hp, HL ratio), except CA. Among these indices, this suggests that TL may covary with some aspect of—but does not completely associate with—individual quality, given that individuals with longer average TL exhibited higher levels of CA.

Higher CA titres are largely considered beneficial as the complement system is an integral part of innate immune defences, initiating adaptive immune memory, and in later life, cellular debris clearance and self-maintenance [70]. Consequently, our result indicates TL might indicate or covary with individual quality with respect to certain immune defensive capabilities. In purple-crowned fairy-wrens, we know that early-life TL [12] and rates of telomere shortening [14] predict life-long fitness and lifespan, in addition to CA also predicting survival [21], so it is conceivable that CA forms an important mediatory link between telomeres and fitness. However in [21], the relationship between CA and fitness was nonlinear and showed individuals with intermediate CA had the greatest survival probability, so it remains unclear whether longer telomeres and higher CA are both attributes of high-quality individuals, or whether some trade-off is at work related to the costs of immune defences. Given its multiple functional roles, CA could also be mitigating telomere attrition against the costly effects of infection or oxidative stress produced by cellular waste, generating directly the telomere-immune covariation that we detected, but with a different causal direction. Our study highlights a potentially important role of the complement system in telomere shortening and fitness. Lastly, the presence of a between- (and possibly not within-) individual effect here also suggests that heterogeneity among individuals is greater than, and probably overrides, the telomere losses experienced by individuals. Individual differences in TL may already exist at birth and processes that determine telomere inheritance ([71]; and e.g. epigenetics; [72]) might then be more important for fitness than ageing, telomerase activity, individual stress-related telomere shortening, or all of the above.

### 4.4. Age-independent effects of telomere length

Age and TL did not explain the same aspects of immune variation. Previously, we found age-related changes in NAbs and HL ratio at the population level [36], but without clear within-individual immunosenescence. Despite TL clearly declining with age (figure 1), there were no relationships between TL and NAbs or HL ratio, either within or between individuals. Instead, there was a clear positive linear relationship with CA, apparently driven by differences between individuals (table 2 and figure 2), even while controlling for age (electronic supplementary material, table S4). Significant age effects were found on NAbs (negative) and HL ratio (positive), consistent with previous results in a similar dataset [36]. Our study shows that TL relates to various aspects of immune function differently and independently of any age-related changes. Consistent with our findings, several studies have shown similar age-independent relationships between TL and immune components [62] or the fitness-related traits potentially underpinned by immune function [73,74]. Aspects of telomere dynamics that are not dictated by age, such as oxidative stress-related telomere losses, should therefore be more pertinent for immune function and/or other fitness-related traits. Age-independence of these between-individual effects thus infers that any potential signal of individual quality provided by TL is more likely a consequence of heterogeneity among individuals in resilience to stressors or other factors that influence telomere dynamics.

## 5. Conclusion

We investigated the relationships between TL and several immune indices while controlling for age-related change. Our results suggest that immune function and TL are both (age-)independent expressions of individual heterogeneity (individual quality), better reflecting variation between individuals than within-individual changes. Integral to survival, the immune system could potentially mediate a link between telomere dynamics and organismal fitness, and explain how telomeres capture individual quality. We show that only CA, which is crucial for innate immunity, is predicted by TL at the between-individual level, consistent with TL as a marker of individual quality. On the one hand, the absence of other relationships here can be taken to indicate that aspects of immune function are unlikely to strongly mediate links between TL and fitness, or explain widespread survival senescence that could be underpinned by immunosenescence—both of which are observed in wild animals [75,76]. On the other hand, given the immense complexity of the immune system, and the plethora of factors that change with age and affect telomeres, that we find the association with CA at all can be taken as an indication that the immune system more generally may well play an important role in mediating the link between TL and remaining lifespan.

## Data Availability

Data and analysis code are available from the Dryad Digital Repository: https://doi.org/10.5061/dryad.v6wwpzgwp [77].

## References

[1] Nussey DH et al. 2014 Measuring telomere length and telomere dynamics in evolutionary biology and ecology. Methods Ecol. Evol. **5**, 299-310. (10.1111/2041-210X.12161) 25834722 PMC4375921

[2] Monaghan P, Eisenberg DTA, Harrington L, Nussey D. 2018 Understanding diversity in telomere dynamics. Phil. Trans. R. Soc. B **373**, 20160435. (10.1098/rstb.2016.0435) 29335374 PMC5784056

[3] Monaghan P, Ozanne SE. 2018 Somatic growth and telomere dynamics in vertebrates: relationships, mechanisms and consequences. Phil. Trans. R. Soc. B **373**, 20160446. (10.1098/rstb.2016.0446) 29335370 PMC5784066

[4] Barnes RP, Fouquerel E, Opresko PL. 2019 The impact of oxidative DNA damage and stress on telomere homeostasis. Mech. Ageing Dev. **177**, 37-45. (10.1016/j.mad.2018.03.013) 29604323 PMC6162185

[5] Reichert S, Stier A. 2017 Does oxidative stress shorten telomeres *in vivo*? A review. Biol. Lett. **13**, 20170463. (10.1098/rsbl.2017.0463) 29212750 PMC5746531

[6] Boonekamp JJ, Bauch C, Mulder E, Verhulst S. 2017 Does oxidative stress shorten telomeres? Biol. Lett. **13**, 20170164. (10.1098/rsbl.2017.0164) 28468913 PMC5454244

[7] Chatelain M, Drobniak SM, Szulkin M. 2020 The association between stressors and telomeres in non-human vertebrates: a meta-analysis. Ecol. Lett. **23**, 381-398. (10.1111/ele.13426) 31773847

[8] Angelier F, Costantini D, Blévin P, Chastel O. 2018 Do glucocorticoids mediate the link between environmental conditions and telomere dynamics in wild vertebrates? A review. Gen. Comp. Endocrinol. **256**, 99-111. (10.1016/j.ygcen.2017.07.007) 28705731

[9] Casagrande S, Hau M. 2019 Telomere attrition: metabolic regulation and signalling function? Biol. Lett. **15**, 20180885. (10.1098/rsbl.2018.0885) 30890069 PMC6451386

[10] Jacome Burbano MS, Cherfils-Vicini J, Gilson E. 2021 Neutrophils: mediating TelOxidation and senescence. EMBO J. **40**, e108164. (10.15252/embj.2021108164) 33880795 PMC8090830

[11] Angelier F, Weimerskirch H, Barbraud C, Chastel O. 2019 Is telomere length a molecular marker of individual quality? Insights from a long-lived bird. Funct. Ecol. **33**, 1076-1087. (10.1111/1365-2435.13307)

[12] Eastwood JR, Hall ML, Teunissen N, Kingma SA, Hidalgo Aranzamendi N, Fan M, Roast M, Verhulst S, Peters A. 2018 Early-life telomere length predicts lifespan and lifetime reproductive success in a wild bird. Mol. Ecol. **28**, 1127-1137. (10.1111/mec.15002) 30592345

[13] Wilbourn RV, Moatt JP, Froy H, Walling CA, Nussey DH, Boonekamp JJ. 2018 The relationship between telomere length and mortality risk in non-model vertebrate systems: a meta-analysis. Phil. Trans. R. Soc. B **373**, 20160447. (10.1098/rstb.2016.0447) 29335371 PMC5784067

[14] Sheldon E et al. 2021 Telomere dynamics in the first year of life, but not later in life, predict lifespan in a wild bird. (10.22541/au.162168440.03821228/v1)

[15] Sudyka J, Arct A, Drobniak SM, Gustafsson L, Cichoń M. 2019 Birds with high lifetime reproductive success experience increased telomere loss. Biol. Lett. **15**, 20180637. (10.1098/rsbl.2018.0637) 30958221 PMC6371901

[16] Tricola GM et al. 2018 The rate of telomere loss is related to maximum lifespan in birds. Phil. Trans. R. Soc. B **373**, 20160445. (10.1098/rstb.2016.0445) 29335369 PMC5784065

[17] Haussmann MF, Heidinger BJ. 2015 Telomere dynamics may link stress exposure and ageing across generations. Biol. Lett. **11**, 20150396. (10.1098/rsbl.2015.0396) 26538535 PMC4685533

[18] Monaghan P. 2010 Telomeres and life histories: the long and the short of it. Ann. N. Y. Acad. Sci. **1206**, 130-142. (10.1111/j.1749-6632.2010.05705.x) 20860686

[19] Sudyka J. 2019 Does reproduction shorten telomeres? Towards integrating individual quality with life-history strategies in telomere biology. Bioessays **41**, 1900095. (10.1002/bies.201900095) 31577044

[20] Jacome Burbano MS, Gilson E. 2021 The power of stress: the telo-hormesis hypothesis. Cells **10**, 1156. (10.3390/cells10051156) 34064566 PMC8151059

[21] Roast MJ, Aranzamendi NH, Fan M, Teunissen N, Hall MD, Peters A. 2020 Fitness outcomes in relation to individual variation in constitutive innate immune function. Proc. R. Soc. B **287**, 20201997. (10.1098/rspb.2020.1997) PMC7735263 33143586

[22] Coppé JP, Desprez PY, Krtolica A, Campisi J. 2010 The senescence-associated secretory phenotype: the dark side of tumor suppression. Annu. Rev. Pathol. Mech. Dis. **5**, 99-118. (10.1146/annurev-pathol-121808-102144) PMC4166495 20078217

[23] Cohen S, Janicki-Deverts D, Turner RB, Casselbrant ML, Li-Korotky HS, Epel ES, Doyle WJ. 2013 Association between telomere length and experimentally induced upper respiratory viral infection in healthy adults. JAMA **309**, 699. (10.1001/jama.2013.613) 23423415 PMC3786437

[24] Arbeev KG et al. 2020 Association of leukocyte telomere length with mortality among adult participants in 3 longitudinal studies. JAMA Netw. Open **3**, e200023. (10.1001/jamanetworkopen.2020.0023) 32101305 PMC7137690

[25] Benetos A et al. 2020 A mechanism for severity of disease in older patients with COVID-19: the nexus between telomere length and lymphopenia. (10.1101/2020.10.01.20205393)

[26] Wang Q et al. 2021 Older biological age is associated with adverse COVID-19 outcomes: a cohort study in UK Biobank. (10.1101/2021.03.20.21254010)

[27] Giraudeau M, Heidinger B, Bonneaud C, Sepp T. 2019 Telomere shortening as a mechanism of long-term cost of infectious diseases in natural animal populations. Biol. Lett. **15**, 20190190. (10.1098/rsbl.2019.0190) 31113307 PMC6548738

[28] Asghar M, Hasselquist D, Hansson B, Zehtindjiev P, Westerdahl H, Bensch S. 2015 Hidden costs of infection: chronic malaria accelerates telomere degradation and senescence in wild birds. Science **347**, 436-438. (10.1126/science.1261121) 25613889

[29] Ilmonen P, Hasselquist D, Langefors Å, Wiehn J. 2003 Stress, immunocompetence and leukocyte profiles of pied flycatchers in relation to brood size manipulation. Oecologia **136**, 148-154. (10.1007/s00442-003-1243-2) 12695901

[30] Tschirren B, Romero-Haro AÁ, Zahn S, Criscuolo F. 2021 Sex-specific effects of experimental ectoparasite infestation on telomere length in great tit nestlings. J. Evol. Biol. **34**, 584-589. (10.1111/jeb.13744) 33226680

[31] Lubbers R, van Essen MF, van Kooten C, Trouw LA. 2017 Production of complement components by cells of the immune system. Clin. Exp. Immunol. **188**, 183-194. (10.1111/cei.12952) 28249350 PMC5383442

[32] Savage HP, Baumgarth N. 2015 Characteristics of natural antibody-secreting cells: natural antibody secretion. Ann. N.Y. Acad. Sci. **1362**, 132-142. (10.1111/nyas.12799) 26104151 PMC4679694

[33] Davis AK, Maney DL. 2018 The use of glucocorticoid hormones or leucocyte profiles to measure stress in vertebrates: what's the difference? Methods Ecol. Evol. **9**, 1556-1568. (10.1111/2041-210X.13020)

[34] Pepper GV, Bateson M, Nettle D. 2018 Telomeres as integrative markers of exposure to stress and adversity: a systematic review and meta-analysis. R. Soc. Open Sci. **5**, 180744. (10.1098/rsos.180744) 30225068 PMC6124068

[35] Shanley DP, Aw D, Manley NR, Palmer DB. 2009 An evolutionary perspective on the mechanisms of immunosenescence. Trends Immunol. **30**, 374-381. (10.1016/j.it.2009.05.001) 19541538

[36] Roast MJ, Hidalgo Aranzamendi N, Teunissen N, Fan M, Verhulst S, Peters A. 2022 No evidence for constitutive innate immune senescence in a longitudinal study of a wild bird. Physiol. Biochem. Zool. **95**, 54-65. (10.1086/717937) 34870562

[37] Teunissen N, Kingma SA, Hall ML, Hidalgo Aranzamendi N, Komdeur J, Peters A. 2018 More than kin: subordinates foster strong bonds with relatives and potential mates in a social bird. Behav. Ecol. **29**, 1316-1324. (10.1093/beheco/ary120)

[38] Roast MJ. 2020 Individual variation in immune function in the purple-crowned fairy-wren (*Malurus coronatus*). PhD thesis, Monash University, School of Biological Sciences.

[39] Davis AK. 2005 Effect of handling time and repeated sampling on avian white blood cell counts. J. Field Ornithol. **76**, 334-338. (10.1648/0273-8570-76.4.334)

[40] Zylberberg M. 2015 Common measures of immune function vary with time of day and sampling protocol in five passerine species. J. Exp. Biol. **218**, 757-766. (10.1242/jeb.111716) 25617452

[41] Hegemann A, Pardal S, Matson KD. 2017 Indices of immune function used by ecologists are mostly unaffected by repeated freeze-thaw cycles and methodological deviations. Front. Zool. **14**, 1-8. (10.1186/s12983-017-0226-9) 28883887 PMC5580329

[42] Campbell TW. 2015 Blood sample collection and preparation in birds. In Exotic animal hematology and cytology, pp. 165-172. New York, NY: Wiley Blackwell.

[43] Eastwood JR, Mulder E, Verhulst S, Peters A. 2018 Increasing the accuracy and precision of relative telomere length estimates by RT qPCR. Mol. Ecol. Resour. **18**, 68-78. (10.1111/1755-0998.12711) 28805012

[44] Criscuolo F, Bize P, Nasir L, Metcalfe NB, Foote CG, Griffiths K, Gault EA, Monaghan P. 2009 Real-time quantitative PCR assay for measurement of avian telomeres. J. Avian Biol. **40**, 342-347. (10.1111/j.1600-048X.2008.04623.x)

[45] Atema E, van Oers K, Verhulst S. 2013 GAPDH as a control gene to estimate genome copy number in great tits, with cross-amplification in blue tits. Ardea **101**, 49-54. (10.5253/078.101.0107)

[46] Roast MJ, Aulsebrook AE, Fan M, Hidalgo Aranzamendi N, Teunissen N, Peters A. 2019 Short-term climate variation drives baseline innate immune function and stress in a tropical bird: a reactive scope perspective. Physiol. Biochem. Zool. **92**, 140-151. (10.1086/702310) 30689489

[47] Andersen CBF, Stødkilde K, Sæderup KL, Kuhlee A, Raunser S, Graversen JH, Moestrup SK. 2017 Haptoglobin. Antioxid. Redox Signal. **26**, 814-831. (10.1089/ars.2016.6793) 27650279

[48] Quaye IK. 2008 Haptoglobin, inflammation and disease. Trans. R. Soc. Trop. Med. Hyg. **102**, 735-742. (10.1016/j.trstmh.2008.04.010) 18486167

[49] Matson KD, Horrocks NPC, Versteegh MA, Tieleman BI. 2012 Baseline haptoglobin concentrations are repeatable and predictive of certain aspects of a subsequent experimentally-induced inflammatory response. Comp. Biochem. Physiol. A. Mol. Integr. Physiol. **162**, 7-15. (10.1016/j.cbpa.2012.01.010) 22316629

[50] Panda S, Ding JL. 2015 Natural antibodies bridge innate and adaptive immunity. J. Immunol. **194**, 13-20. (10.4049/jimmunol.1400844) 25527792

[51] Matson KD, Ricklefs RE, Klasing KC. 2005 A hemolysis–hemagglutination assay for characterizing constitutive innate humoral immunity in wild and domestic birds. Dev. Comp. Immunol. **29**, 275-286. (10.1016/j.dci.2004.07.006) 15572075

[52] Genovese KJ, He H, Swaggerty CL, Kogut MH. 2013 The avian heterophil. Dev. Comp. Immunol. **41**, 334-340. (10.1016/j.dci.2013.03.021) 23583524

[53] Sharma JM. 1991 Overview of the avian immune system. Vet. Immunol. Immunopathol. **30**, 13-17. (10.1016/0165-2427(91)90004-V) 1781153

[54] Krams I, Vrublevska J, Cirule D, Kivleniece I, Krama T, Rantala MJ, Sild E, Hõrak P. 2012 Heterophil/lymphocyte ratios predict the magnitude of humoral immune response to a novel antigen in great tits (*Parus major*). Comp. Biochem. Physiol. A. Mol. Integr. Physiol. **161**, 422-428. (10.1016/j.cbpa.2011.12.018) 22245489

[55] R Core Team. 2020 R: a language and environment for statistical computing. Vienna, Austria: R Foundation for Statistical Computing. See https://www.R-project.org/.

[56] Lin J, Smith DL, Esteves K, Drury S. 2019 Telomere length measurement by qPCR – summary of critical factors and recommendations for assay design. Psychoneuroendocrinology **99**, 271-278. (10.1016/j.psyneuen.2018.10.005) 30343983 PMC6363640

[57] Kuznetsova A, Brockhoff PB, Christensen RHB. 2017 lmerTest Package: tests in linear mixed effects models. J. Stat. Softw. **82**, 1-26. (10.18637/jss.v082.i13)

[58] van de Pol M, Wright J. 2009 A simple method for distinguishing within- versus between-subject effects using mixed models. Anim. Behav. **77**, 753-758. (10.1016/j.anbehav.2008.11.006)

[59] Dantzer B, Fletcher QE. 2015 Telomeres shorten more slowly in slow-aging wild animals than in fast-aging ones. Exp. Gerontol. **71**, 38-47. (10.1016/j.exger.2015.08.012) 26348426

[60] von Zglinicki T. 2001 Telomeres and replicative senescence: is it only length that counts? Cancer Lett. **168**, 111-116. (10.1016/S0304-3835(01)00546-8) 11403914

[61] Forsyth NR, Wright WE, Shay JW. 2002 Telomerase and differentiation in multicellular organisms: turn it off, turn it on, and turn it off again. Differentiation **69**, 188-197. (10.1046/j.1432-0436.2002.690412.x) 11841477

[62] Beirne C, Waring L, McDonald RA, Delahay R, Young A. 2016 Age-related declines in immune response in a wild mammal are unrelated to immune cell telomere length. Proc. R. Soc. B **283**, 20152949. (10.1098/rspb.2015.2949) PMC4810837 26888036

[63] Fairlie J, Holland R, Pilkington JG, Pemberton JM, Harrington L, Nussey DH. 2016 Lifelong leukocyte telomere dynamics and survival in a free-living mammal. Aging Cell **15**, 140-148. (10.1111/acel.12417) 26521726 PMC4717268

[64] Olsson M, Geraghty NJ, Wapstra E, Wilson M. 2020 Telomere length varies substantially between blood cell types in a reptile. R. Soc. Open Sci. **7**, 192136. (10.1098/rsos.192136) 32742684 PMC7353983

[65] Reichert S, Criscuolo F, Verinaud E, Zahn S, Massemin S. 2013 Telomere length correlations among somatic tissues in adult zebra finches. PLoS ONE **8**, e81496. (10.1371/journal.pone.0081496) 24349076 PMC3857187

[66] Daniali L, Benetos A, Susser E, Kark JD, Labat C, Kimura M, Desai KK, Granick M, Aviv A. 2013 Telomeres shorten at equivalent rates in somatic tissues of adults. Nat. Commun. **4**, 1597. (10.1038/ncomms2602) 23511462 PMC3615479

[67] Kimura M, Gazitt Y, Cao X, Zhao X, Lansdorp PM, Aviv A. 2010 Synchrony of telomere length among hematopoietic cells. Exp. Hematol. **38**, 854-859. (10.1016/j.exphem.2010.06.010) 20600576 PMC3142672

[68] Haussmann MF, Marchetto NM. 2010 Telomeres: linking stress and survival, ecology and evolution. Curr. Zool. **56**, 714-727. (10.1093/czoolo/56.6.714))

[69] Casagrande S, Stier A, Monaghan P, Loveland JL, Boner W, Lupi S, Trevisi R, Hau M. 2020 Increased glucocorticoid concentrations in early life cause mitochondrial inefficiency and short telomeres. J. Exp. Biol. **223**, jeb222513. (10.1242/jeb.222513) 32532864

[70] Ricklin D, Hajishengallis G, Yang K, Lambris JD. 2010 Complement: a key system for immune surveillance and homeostasis. Nat. Immunol. **11**, 785-797. (10.1038/ni.1923) 20720586 PMC2924908

[71] Vedder O, Moiron M, Bichet C, Bauch C, Verhulst S, Becker PH, Bouwhuis S. 2021 Telomere length is heritable and genetically correlated with lifespan in a wild bird. Mol. Ecol. mec.15807. (10.1111/mec.15807) 33460462

[72] Bauch C, Boonekamp JJ, Korsten P, Mulder E, Verhulst S. 2019 Epigenetic inheritance of telomere length in wild birds. PLoS Genet. **15**, e1007827. (10.1371/journal.pgen.1007827) 30763308 PMC6375570

[73] Bize P, Criscuolo F, Metcalfe NB, Nasir L, Monaghan P. 2009 Telomere dynamics rather than age predict life expectancy in the wild. Proc. R. Soc. B **276**, 1679-1683. (10.1098/rspb.2008.1817) PMC2660992 19324831

[74] Pauliny A, Wagner RH, Augustin J, Szép T, Blomqvist D. 2006 Age-independent telomere length predicts fitness in two bird species. Mol. Ecol. **15**, 1681-1687. (10.1111/j.1365-294X.2006.02862.x) 16629820

[75] Nussey DH, Froy H, Lemaitre JF, Gaillard JM, Austad SN. 2013 Senescence in natural populations of animals: widespread evidence and its implications for bio-gerontology. Ageing Res. Rev. **12**, 214-225. (10.1016/j.arr.2012.07.004) 22884974 PMC4246505

[76] Peters A, Delhey K, Nakagawa S, Aulsebrook A, Verhulst S. 2019 Immunosenescence in wild animals: meta-analysis and outlook. Ecol. Lett. **22**, 1709-1722. (10.1111/ele.13343) 31321874

[77] Roast MJ, Eastwood JR, Aranzamendi NH, Fan M, Teunissen N, Verhulst S, Peters A. 2022 Data from: Telomere length declines with age, but relates to immune function independent of age in a wild passerine. Dryad Digital Repository. (10.5061/dryad.v6wwpzgwp) PMC9043702 35601455

